# Death literacy: Tensions between theory and practice

**DOI:** 10.1177/26323524261429985

**Published:** 2026-03-24

**Authors:** Jennifer Rogerson, Daniella Holland-Hart, Michelle Edwards

**Affiliations:** 1 Division of Population Medicine, Marie Curie Research Centre, Cardiff University, UK

**Keywords:** death literacy, end-of-life, palliative care, policy

## Abstract

Death literacy is a burgeoning field in palliative care research and the social sciences, exploring people’s knowledge, skills, experiential learning and social action as these pertain to death and dying. Death literacy is not described or advocated in UK policy or guidelines explicitly, yet this essay shows that it is mentioned implicitly, and these implicit definitions and uses are explored. In this critical essay, the authors draw on a series of examples from UK-based policy and guidelines to describe the ways death literacy is implicitly articulated in the material. In doing so, the essay draws attention to the ways death literacy is understood in policy and guidelines, the ways death and talking about death are framed, and the ways that nuanced contextual accounts of death literacy are critical to developing this branch of study further. By looking at the ways death literacy is framed in policy through the lenses of time, taboo and how death is discussed (or not), this essay develops an account of the tensions and gaps between theory and practice. Recommendations to address these gaps include discussions centred on the timing of conversations about death and dying, and establishing a benchmark for what is discussed and constitutes ‘death literacy’ in public discourse. As such, the essay contributes to the growing body of literature on death literacy in the United Kingdom.

## Plain language summary

Death literacy is a term that is being used in policy, social sciences and death studies to understand and explore the ways people know about death and dying, often based on their experience. Death literacy has not been widely or empirically studied in the UK, and so the policy and guidelines in the UK do not explicitly draw on the term. This essay therefore describes how policy and guidelines implicitly talk about death literacy. These descriptions enable the authors to begin offering ways death literacy might be understood and defined in policy. The authors also identify where there are gaps between how death literacy is understood, and how it is practiced.

## Introduction

Death literacy has emerged as a term used to describe knowledge and skills relating to end-of-life, often in which people come to be death literate through experiences and practice-based care. The concept has been described as encompassing four key components: knowledge, skills, experiential learning and social action.^1^ In the United Kingdom, the death literacy index (DLI)^2^ has been used to determine levels of death literacy.^3^ The DLI was developed in Australia as a ‘useable measure for death literacy’ that can be used to assess interventions in individual and community settings related to death and dying.^2,4^ Globally, other countries have validated the DLI; including Turkey,^5^ Sweden^6^ and southern China.^7^ The DLI offers a useful benchmark and invites nuanced, contextual accounts of how death literacy might materialise and be shaped: there are myriad ways ‘palliative care’ can be understood, alongside connotations with the term, for example. These myriad framings need to be explored.

The subject of death literacy draws attention to and encompasses multiple aspects of navigating end-of-life and the ways it is approached. Increased medical technologies, legal and litigious orientations to care, policy and economic directives, and emphasis on the autonomous individual, define and shape death contemporaneously.^8^ A model in which knowledge of and decision-making approaches to death are emphasised, presents a stark contrast to death studies in which importance is placed on funerary rites and the rituals associated with death. Medical technologies – that to some degree – control the end-of-life and *how* a person dies, have remodelled how death is managed and communicated, shifting what is known about death and EoL care, as well as the experiences of those able to access more or less medical care and attention.^9^ These changes are all aspects that can be explored through the lens of different literacies relating to end-of-life.

Responding to death as individuals and as societies requires coming to know about death and dying processes; these ways of knowing are varied and multiple. The term ‘death literacy’ is not widely used in public and healthcare spheres in the United Kingdom and similarly in other countries but has bearing where an ageing population, the promotion of EoL care in community settings, and emphasis on individual choice-making, means being knowledgeable and death literate is necessary to EoL care objectives. Exploring death literacy as a concept is therefore relevant to the United Kingdom: as an emergent term, there is room to explore how death literacy is discussed conceptually in UK literature. This essay describes how policy and guidelines implicitly talk about death literacy and how these descriptions offer ways death literacy might be interpreted, understood and defined in policy, and the consequences of these interpretations.

## Death literacy and the UK context

As a burgeoning field, the literature on death literacy in the United Kingdom is largely implicit; there is an absence of specific references to death literacy within policies and medical guidelines focused in the United Kingdom. Yet, it is evident that death literacy is tacitly apparent in these materials. This essay provides a series of snapshots from a range of academic literature, policy and medical guidelines that highlight the formal approaches to discussing and planning for death in the United Kingdom. We draw on literature and policies that focus on the ways aspects of death, such as end-of-life care and palliative medicine, are politically and legally constituted and framed, with an implicit expectation that people are knowledgeable about death and dying. By exploring the ways public knowledge and literacy are built into policy and guidelines (or not), we establish the contexts, tensions and gaps across a range of literature on death literacy. In doing so, this essay contributes to developing a conceptual account of death literacy, providing nuance to the context and term, and the types of knowledge, information, awareness, equalities and conversations that are imagined in a death literate society. We also explore the tensions that materialise between death literacy theory and practice.

Currently, many UK government policies emphasise the importance of encouraging people to die at home.^10^ Dying at home is less expensive to The State, it ensures beds in hospitals are not used for people without treatment options and is often cited as people’s preferred place of death.^11^ Knowledge of death has previously been associated with knowing at an individual level, and individual decision-making is emphasised^12^; dying at home, however, draws attention to the need for community-wide death literacy. Operationalising the preference to die at home means providing people with both the knowledge and means to facilitate home death and planning accordingly. Yet being knowledgeable about death and dying, or talking about it in the United Kingdom, is not well established and is largely relegated to discussions about advance care planning (ACP).

The Public Attitudes to Death and Dying UK study^13^ examined the aspects of approaches to dying, including knowledge and literacy of terms used in EoL care and planning. The results indicated that terminology related to death and dying such as palliative care was not widely used or understood. Interestingly, 51% of people surveyed said that people do not talk about EoL enough and its associated care, yet 84% of participants indicated that there is nothing to stop them talking to family about EoL, but most had not actually done so. The finding indicates a series of interpretations: the first is that people wish to talk about death and dying, and factors outside of comfort in discussing death are preventing communication, and those factors need to be determined. The second interpretation is that communication may be hoped for, but lack of knowledge of EoL care and planning prevents people from engaging death discussions.

Finally, it is not clear what *kinds* of EoL conversations 84% of people did not have a problem discussing. People may be comfortable discussing death in society in a generalised way but communicating about their own mortality, death and the impact it will have, may not be comfortable to people. It is therefore necessary to develop a closer examination of the kinds of death conversations people understand to constitute death literacy, as well as the kinds of communication that would be useful to death literacy measures, policies and guidelines. These terms and kinds of communication are central to the information repertoire necessary to navigate EoL, particularly those being cared for or caring in the community. Death literacy is therefore a kind of knowledge that extends beyond the purview of care professionals and calls for individuals to have a generalised competence towards EoL care.^6^

Regularly nested within discussions on death and dying are expectations towards community involvement in EoL care, and self-responsibilisation when it comes to EoL. Self-responsibilisation is a term used to describe the ways that emerging neoliberal forms of governance operate, in which emphasis is placed on empowerment and independence of individuals.^14^ Sets of ideals involve a shrinking state mandate and a widening of responsibilities placed on the individual.^15^ As Freeman and Napier^16^ explain, within this context, autonomous actors are often entangled with social and personal dependencies and duties. Here, duties and choice may in reality not be realised or possible due to social practices, political pressures and socio-economic restrictions including the remit of social and healthcare systems or a lack of financial means at end-of-life.^17^

Such forms of self-responsibility are found in medical practise where a move away from paternalistic medical practise, and towards patient choice establish a mode in which individuals become responsible for themselves. Strained medical services make the transition from reliance on medical services to self-responsibilisation and community responsibility more pressured for patients and carers.^15,18^ A knowledgeable, death literate society is critical to such a move but asks about the place of community and relational practises of care in social life, and how these might be grown. Questions of power and inequalities also emerge, where some are enabled to engage more with self-responsibilisation objectives than others. Questions of responsibility, the individual and community therefore ask how death literacy might materialise in these contexts.

The materialisation of death literacy in these settings can make for tensions in both expectations and possibilities for community action and the place of individuals. Community endeavours such as Compassionate Communities are increasingly espoused by governments as a means of enabling community-based care. However, there are gaps in provision as well as clear pathways to facilitating these programmes. Likewise, community-focused activities might be more easily initiated and relevant within communities with more financial and social capital to provide care within their own communities. In other cases, communities might have the connections and impetus to care for one another but avoid necessary medical support due to a feeling of disconnect with healthcare services, as well as health inequalities barriers. We explore these kinds of tensions.

## Developing death literacy

### Health literacy

How a person knows about their well-being and accessing healthcare relates to their understandings about health, and literacy levels. Kickbusch^19^ explains literacy as a complex set of abilities to understand and use the dominant symbol system of a culture for personal and community development. Importantly, the need and demand for these abilities vary in different societies. There are various kinds of literacy – quantitative, scientific, technological, cultural literacy, and all forms of literacy are needed to function in one’s social world at different times and contexts.

Health is a critical aspect of how and whether people can live in and navigate social life and death. Health and disease exist within social and cultural worlds; knowledge of health must be part of how people navigate their daily lives. As people engage with more complex health systems, it is necessary to recognise that relationships in healthcare are laden with power and politics, and shaped by history.^20,21^ Likewise, Nutbeam^22^ points out that literacies are not individual endeavours: environments, economic conditions and state-crafted incentives and support are necessary to develop health literacies, and therefore death literacy. It is this, community-involved emphasis, that is both critical to the imagining and practise of death literacy as an outcome, and death literate societies.

### Death education

Death education constitutes a body of literature that is largely based on how death has moved in and out of society as an acceptable subject and theme. As a discipline, death education attempts to draw attention to death in public and scholarly life. Fonseca and Testoni^23^ describe the changes of modern societies, in which many aspects of life became invisible and unspoken, death being one such event.^24^

Historically, death, alongside birth and other ‘fleshy’ aspects of life was in evidence in the everyday and was visible, occurring largely at home and within family and community spaces and care.^25^ Death in this context was neither necessarily lonely nor unknown to the living. The transition from death as a family-community experience to one managed professionally is perhaps one of the greatest changes that has affected the ways people contemporaneously handle, understand and know about death.^24,26^

As death became more ‘sanitised’ and it became apparent to health professionals and those supporting death that the public’s engagement, comfort and knowledge of death was becoming absent, researchers and educators attempted to draw attention to death, encouraging discussions, research and education on death.^27^ ‘The Death awareness movement’ or ‘death education’ became the terms used to describe the transition of researchers to bring death back into social and public awareness.^28^ Death education, in this sense, is therefore the knowledge and experience a person acquires over a lifetime. As a part of cultural life, death education is partly existential, asking people to recognise and acknowledge that their lives are limited. As scholars of death education recognise that family and community are the first source of death education, the formalised study and crafting of a discipline of ‘death education’ is precarious in its informality.^28–30^ In attempts to formalise death education to some degree, the discipline includes the articulation of goals centred on death and dying, content and perspectives on death and education, teaching methods and evaluation.^28^

As death education grew as an area of study, more nursing schools and palliative care professionals realised the lack of knowledge on death, amongst both health professionals and publics; emphasis on death education increased.^31^ As a result, government policies and directives exist that focus specifically on palliative and EoL care. Death literacy has commonalities with death education, attempting to define and quantify knowledge and literacy, and who has it – professionals and/or publics – centred on death and end-of-life care.

### Decision-making: The role of risk and technologies

Literacies are informed by the ways people approach decision-making, health and illness. Douglas and Wildavsky^32^ explain that the ways people come to make decisions are informed by how they understand risk. There are differing views for different people in what constitutes risks, what is risky, how risky something is and what to do about it. Death literacy is shaped by risk, whereby people are asked to make decisions and decide on care pathways based on information and risks presented, as well as cultural knowledge of risks and the varying impacts such decisions could have on themselves, their families and wider support networks. Indeed, whether one has a support network or community influences risk in terms of care and support available to people outside of hospital, and whether people are asked to make decisions for care on their own or within a community and family support.

Kaufman et al.^9^ describe the ways that reduced risks of biomedical procedures produce changing approaches and attitudes towards life extension and ‘old age’. The language and logic of risk, Kaufman et al.^9^ argue, influences decisions to intervene as interventions are experienced as incremental and thus, unremarkable, crafting the pursuit of an open-ended future. How people understand death, the life cycle and how death is approached is therefore shaped by shifting parameters of death and expectations of death due to medical technologies. The reduction of risk of medical intervention means that more knowledge of intervention, technologies and the medical management of life-limiting illnesses and death is required of people. Kaufman et al.^9^ write of the emergence of risk as a contemporary category of knowledge: bodies, including dying bodies, are increasingly disciplined and ‘risk factors’, medical surveillance, cultural framings of health maintenance, social and familial obligations, constitute taken-for-granted aspects of how death and EoL decisions and practices are considered.^33^ Clarke et al.^34^ describe strategies inside and outside clinic spaces aimed at rearranging individuals as active partners in the pursuit of health, and participants in their own end-of-life care.

Indeed, death studies scholars’ comment on the movement of end-of-life care and death from home to hospital in contemporary social life, and how this spatial movement of death changed how much is known about death based on its current invisible quality, because it is largely outside of everyday life.^25^ Yet, increased technologies and interventions require different and increased forms of knowledge, built on notions of risk, life-extension technologies and the decision-making processes called forth with more interventions available and risks that must be weighed – forming aspects of contemporary death literacies.

In the rest of this essay, we offer snapshots of policy and medical guidelines as ways of thinking through the considerations raised above, and how death literacy is indirectly approached by the UK State (mainly England, rather than all four nations), and through medical mandates. It is our hope that these insights contribute to the growing body of work on death literacy.

## Death literacy: Tensions in the guidelines

### Time, timing and death literacy: When to know about death and dying

The NHS is required to offer palliative and end-of-life care as a statutory requirement of the Health and Care Act.^35^ Therefore, there is a differentiation established in law, between naming and defining care for those in the end-of-life period and those who are receiving other kinds of healthcare. According to the General Medical Council^36^ guidelines, patients are defined as approaching the end-of-life when they are likely to die within the next 12 months of their lives. The 12-month timeframe is important because it indicates two ways of viewing EoL. The first is that in medical terms, EoL is given temporal boundaries. Secondly, it also introduces a specific kind of support focus – end-of-life care.

There are therefore two kinds of boundaries in EoL care provision: legal and clinical. While defining EoL is practical from a clinical perspective, in terms of care provision over the life course, the timeframe crafts a framing of EoL in which both acknowledgement of, discussions and care are relegated to a particular part of the life cycle: the last phase of life. In terms of linear framings of time progression, legally and clinically, EoL as a kind of care is *excluded* from the wider life cycle. In other words, until one is defined as having a progressive, life-limiting illness or approaching death within a year, EoL and its associated discussions are not necessarily deemed clinically relevant in wider life. The point draws attention to whether these time-framings are fruitful to wider conversations across life, what other temporal possibilities might look like and how the definition of 1 year assumes a singular, limiting trajectory for how people approach end-of-life as a phase.

The NICE *End of life care for adults: service delivery*^37^ evidence review offers an example of these timing considerations. In the main findings, 13 themes were identified, of which 9 related to literacy, knowledge and understanding, and almost all the main findings in the NICE evidence review could be defined as aspects of death literacy. Specifically, ‘point 12’ in the findings indicated challenges relating to timing and an acknowledgement of dying and willingness to discuss death:

Planning for one’s own dying and death was not something that people with lung cancer reported having discussed, except in relation to the practical arrangements following death. They preferred to focus on living in the present by ‘carrying on as normal’ whilst they still felt reasonably well, seeking to postpone facing death until the time came. (NICE^37^, p. 20)

This point speaks to the critical conflation of temporality and death literacy, and what is discussed in terms of death over the life course. The finding indicates that people are challenged by a weighing up of ‘living in the present’ and enjoying life while well, leaving death discussions to the ‘end’; and pragmatically preparing for and acknowledging the place of death in life.

The *What matters most charter*^38^ speaks to literacy and ACP, and their relationship to time. For example, point three in the charter hoped to be ‘supportive of individuals making their choices for their own living well, *up to and including the end part of their life*’.^38^ Yet, the charter indicates that people should have an early repertoire of end-of-life literacies to make those choices. Part of conceptualising death literacy means asking, *how* advanced might ACP be expected to be: ACP is currently associated with a time when end-of-life is imminent, but it could potentially begin decades before the end-of-life period, enabling more informed conversations. Yet, *Universal principles for ACP*^39^ indicated that reviewing patients’ ACP is ‘triggered by events, such as a deterioration in their condition, an acute admission to hospital or a significant conversation with those important to them’,^39^ that is, when death becomes imminent; a timing that does not align with making people death literate over the life course.

*Choice in end-of-life care: a government response*^40^ also emphasised the place of timing discussion and information sharing at the EoL stage. The document calls for ensuring that the right people, knowledge and skills are made available to offer high-quality care. Conversations regarding EoL are marked as important:

We will continue to work closely with our voluntary sector partners including on specific projects to improve end-of-life care in hospital and out-of-hospital settings, promote a national conversation about death and dying and develop local volunteer networks. (NHS Finance and Operations/NHS Group/NHSCS/17189^40^)

While a national conversation about death and dying is identified as a priority, it is not clear what *kinds* of conversations and importantly, *when* conversations should begin. There are varying timeframes possible: throughout life, as part of people’s social and healthcare worlds over a lifetime; when a friend, family member or self is diagnosed with a life-limiting illness; or in the last year of life. The implication of national conversation also draws attention to differences between public health initiatives and supporting people to have interpersonal discussions. It is evident that there is a gap between identification of a need for death conversations and defining of these conversations and their timeframes. *Choice in end-of-life care: a government response*^40^ indicates that underpinning care is:

The ability to have the most difficult conversations about death, dying and the course of a disease or condition. . .There has been a growing recognition over recent years that to achieve high quality end of life care in all settings there must be a concerted effort to improve the education and training in end-of-life care-specific issues that all doctors, nurses and other health and care staff receive. (NHS Finance and Operations/NHS Group/NHSCS/17189^40^)

Conversations about dying are framed from the goal-oriented perspective of ensuring patients receive good care. Yet little mention is made of public education campaigns and therefore, an unequal balance of who has knowledge – public versus professionals emerges. The point draws attention to associated challenges related to acknowledging dying and willingness to discuss death across the life course, or only at diagnosis.

Attempting to undo the framework of literacy beginning only at diagnosis, the Scottish Partnership for Palliative Care produced *A road less lonely*^41^ which emphasised the importance of death education support in schools and facilitating lessons with a specific focus on death as part of the life cycle, as an integral part of equipping children to deal with loss and change. Achieving this vision included improving staff confidence, developing a school bereavement policy and death education: facilitating lessons with a specific focus on death as part of the life cycle.^41^ They suggested creating a charter for a ‘good end-of-life’:

This would involve a programme of work to engage with the public, media, and health and social care professionals to produce honest and accessible information about the support people can expect to receive as they approach the end of life. (Patterson et al.^41^)

Death literacy was therefore presented as a process that occurs when knowing about death is incorporated temporally into wider social life, including practical and didactic approaches incorporated into school curricula. Accepting that death is ‘part of life’, to be incorporated from early life, was operationalised as a method for ensuring knowing, good death management and a good death experience, that could be coupled with experiential learning: ways of becoming death literate. The question of who should be the focus in developing death literacy is raised. Professionals, all publics, those in the dying phase, or those caring and knowing those in the dying phase are all groups made possible here. These documents begin to offer critical points for death literacy definitions including *who*, *what* and *when* death literacy might entail.

Kaufman^42^ argues that the future, when it comes to end-of-life care, is ‘colonised’. By this, she means that time is collapsed into the present through both knowledge and the known (and unknown) unavoidability of risks, and the associated mechanisms for their calculation and assessment. In other words, time, via the work of technologies and medical interventions, organises life-planning strategies, risk awareness and prevention practises. These strategies and forms of knowledge constitute the embodied and lived ways that people experience and navigate EoL and knowledge of death. Time is clearly critical to establishing conversations and literacies, and questions of whether death literacy should be part of wider life are raised; it also asks how communication and knowledge acquisition take place, how they connect with time, and with whom.

### Talking about death: Taboo, denial and conversation

So far, we have discussed the ways time accounts for and shapes definitions of death literacy. In this section, we turn to the people considered in death literacy debates, and expectations relating to their conversations. Questions of professional and public versions of knowledge offer an entry point. The *Ambitions for palliative and end of life care: A national framework for local action 2021–2026*^43^ offered by NHS England focuses on staff and professional literacy: ‘too often the employers of health and care professionals have not acted systematically to help their staff avoid the debilitating effects of burn out, avoidance or helplessness resulting from lack of education, training and support’. The point is critical: staff who are not supported and knowledgeable cannot offer care to others and themselves. Related, but unsaid in the *Ambitions*^43^ framework, is the publics and communities doing unpaid end-of-life care work. It is recognised that a lack of knowledge and skills can lead to burn out for professional staff, but as part of a wider death literacy debate, the question of who cares and how much they know becomes apposite, particularly as informal carers may experience the same risk of burnout as professionals in a caring capacity. It begins to emerge that *who* death literacy relates to, *who* receives knowledge, and the power dynamics of information exchange form gaps in death literacy parameters, particularly as community-based care is set to increase. These gaps connect with conversation and whether talking about death happens (or should happen) at all.

The *Ambitions* framework,^43^ mentioned above, drew attention to ‘the taboo that many people feel when it comes to talking about dying, death and bereavement and facing up to their own mortality and that of the people important to them’. The framework indicated: ‘we cannot defeat death. However, we can change the way we talk about dying, death and bereavement and prepare, plan, care and support those who are dying and the people who are close to them’. Death is presented as a fact of life (and a problem that cannot be defeated) which is made easier to engage with effectively when one knows about and acknowledges that fact.^44^ Knowledge is power in this rendition; knowledge is also established as a tool for better outcomes.

Likewise, the *Strategic framework for action on palliative and end of life care*,^45^ the Scottish EoL policy document, argued for better understanding of people’s health literacy needs and creating more openness about death. Health literacy and death literacy are linked here but their categorical separation (death as separate from general health literacy) acts to reinforce the broader challenge that the *Strategic framework for action on palliative and end of life care*^45^ itself tries to tackle by bringing death conversation into the life cycle: death is framed as a fact (and problem) that can be solved with good care, that can be offered when the barriers of ‘death as a taboo’ are removed. This point was further emphasised in the Government response for the House of Commons Health and Social Care Committee: *Assisted dying/assisted suicide*.^46^ The response to the recommendation for a national strategy for death literacy (which was not defined) indicated that the government: ‘[does] not plan to establish a national strategy for death literacy. However, the Government’s inclusion of palliative and end-of-life care in wider strategies will continue to drive the conversation and reduce the taboos associated with death and dying’.^46^ EoL care strategies and reducing taboos were framed as equivalent to a death literacy strategy in this rendition. The ‘death as part of the life cycle’ rhetoric established a temporal stretching of death literacy in which death as a topic to be broached, is incorporated into wider social life and broadens its defining terms and purpose.

Reiterating this point, the *Strategic framework for action on palliative and end of life care*^45^ suggested that ‘meaningful conversation’ acts as a method for ‘breaking down barriers’ for engaging with death as a taboo. Knowledge, conversation and meaningfulness are methodologically linked in these documents, and the material asks what the relationship is between discussion, information and the sharing of information relationally and across populations. In a slightly different but connected vein, the *Strategic framework*^45^ explains challenges to meaningful conversations in the context of death as the result of:

A ‘death-denying culture’ and the medicalisation of death. An entire generation has come to expect that all aspects of dying will be taken care of by professionals and institutions, potentially undermining personal and community resilience in coping with death, dying and loss as part of the ‘cycle of life’. (Scottish Government^45^)

A connection was established between the medicalisation of death, death-denial and less community involvement. It is remedied by finding: ‘opportunities to work with social and mass media and wider publics, across educational establishments, business, faith groups, community organisations and creative industries’, thus broadening the parameters and actors in community.

‘Meaningful’ death discussion, the opposite of a ‘death-denying culture’ is therefore imagined and crafted in the context of a community-based, non-medicalised and ‘death-open’ society (see Seymour^47^ for a problematisation of this binary and see Zimmerman^48^ for an analysis of the term ‘denial’ as it is used in EoL care). In the *Strategic framework*,^45^ death as an inevitable event that can be discussed ‘openly’ and ‘widely’ constitutes versions of ‘meaningful’ and engages with the social frame that to ‘speak is to heal’ and that ‘owning up’ to truths is always better. The framing of the importance of effective decision-making, that is premised on open discussion, exists in tension with the taboo of death in which no discussion happens.

## Consolidating theory and policy in practice

While versions of death literacy have been defined by Noonan et al.^1^ and in studies applying and validating the DLI in specific country studies,^2–4,6,7^ these studies have focused on what people currently know about death. Further exploration is needed to identify what kinds of knowledge identified in the DLI mean to individuals, and how they land in everyday social worlds. For example, willingness to discuss death does not reveal what *kinds* of death discussions people are referring to when answering questions.

The snapshots in this essay indicate that knowledge of certain procedures, terms and aspects of end-of-life and palliative care were identified as important in policy making. Yet the policies neither clarified those meanings nor explored what they might mean to general publics, or how they could be applied. For policy to be effective, it is critical to differentiate between what knowledge and terms mean to people, the perceptions and connotations of terms to different people and the myths and beliefs associated with aspects of medical procedures, terms and death itself. Such a focus draws attention to the need for qualitative development of nuanced meanings of what death literacy might be and what it can mean in different contexts and life phases.

Much of the literature described in this essay grapples with an unacknowledged mismatch between how people might become death literate at different life stages. Current policy and medical guidelines indicate that versions of death literacy are only formally introduced from a policy, legal and clinical perspective at the point a person is diagnosed with a life-limiting, terminal illness or are thought to be in the last year of their lives. Yet, many of the discussions required at these end stages are medically, ethically and emotionally complex. Patients and their families are implicitly expected to build and grow a wide body of knowledge and literacy in a reasonably short period of time to fully grapple with clinical discussions and decisions. The DLI studies have shown that people who have cared for someone at the end-of-life necessarily have a better level of death literacy than others.^1,6^ This evidence points to the ways that acquiring experiential and information-based knowledge over the course of caring for another person, directly impacts one’s own death literacy. For people who are diagnosed as moving into the dying phase of their life, who have never been involved in end-of-life care previously, their death literacy may be inadequate for the complex level of information and knowledge-based decision-making to come. It is pertinent to grapple with questions of when death literacy starts and when it *should* start.

## Recommendations and considerations for future theory and practice

Alongside timing and when death literacy takes place, the question of who death literacy is aimed at is important in relation to timings and when learning is initiated. Death literacy could begin when people are at school, as a life skills training. It could be set as a wider existential ongoing discussion over a lifetime that asks people to engage with the meaning of their lives and the inevitability of death. In this regard, death literacy asks how individuals and the social worlds they inhabit value life, and how mortality is ignored and/or confronted and at what phases in life mortality should be addressed individually, socially and medically, or if at all.

This kind of death literacy opens possibilities in terms of what health literacy and campaigns for knowledge-making might look like. Death literacy calls into question the boundaries instantiated legally and clinically around end-of-life and asks what EoL care discussions might come to look like with a broader death literacy built over a lifetime.

It is also important to engage negotiations of knowledge: publics and professionals will have different knowledge, levels of literacy and sources of information. Power dynamics can emerge in how knowledge is shared (including growing digital literacy inequities), who shares with whom and how knowledge is made. As Buch^49^ writes: ‘arrangements of care in later life are deeply enmeshed in the everyday relations that constitute lived experiences of global political-economic shifts’. Death literacy, as an aspect of care, therefore, reflects the broader frame in which it is situated. Expectations towards decision-making and literacies mirror the ways that EoL care is contemporaneously modelled on moving EoL care into community settings, in which individuals and their communities are responsible for their own experience. Therefore, ensuring both professionals and publics are equipped with relevant literacies is germane.

Language, dialogue and conversation are key themes, and it is pertinent to ask how conversation fits with death literacy. Having ‘open’ death conversations forms a significant part of how death literacy is framed in the UK policy and guidelines. Conversation is presented as a method for ensuring good care and a good death via becoming literate. EoL policy commits the NHS to providing palliative care universally, but the frame that improving dying through open conversation, does not necessarily apply to all, and requires sensitivity to a range of approaches.^50,51^ Yet, it is worth considering whether conversation operates as a method for making people death literate or if conversation is an outcome and aspect of being death literate. It is likely that the method and the outcome share overlaps. The shared aspects of conversation, however, highlight the challenges for death literacy: people need to be able to have discussions to be death literate, but need to be death literate to enable discussions. The inherently interpersonal quality of conversation also points to emphasis on both individual and relational care practices.

## Concluding remarks

The tension in death literacy between knowing, and the actual practice of establishing literacy in community spaces, draws attention to gaps in the medical guidelines and the ways policy implicitly addresses the importance of death literacy. Thinking about death literacy through the lenses of *when*, *who* and *what* to develop meaningful accounts of what knowledge, skills, information and literacy might look like in relation to death is critical. More so, it is essential to think through what this kind of knowing might look like in practice. Addressing death literacy as a form of knowing for publics and healthcare professionals is vital. Power dynamics and questions of health inequalities come to bear on how one can become death literate, and whether death ‘literacy’ as a term is best suited in all contexts. These interpretations create space for qualitative research accounts to bolster the current DLI tools, to further and deepen understandings of death literacy, and the contexts and social worlds that make varying kinds of death literacy both possible and practicable.
